# Analysing substitutions in recent World Cups and European Championships in male and female elite football – influence of new substitution rules

**DOI:** 10.5114/biolsport.2024.134755

**Published:** 2024-02-12

**Authors:** Xiaobin Wei, Yang Shu, JiaJun Liu, Paweł Chmura, Morten B. Randers, Peter Krustrup

**Affiliations:** 1 School of Strength and Conditioning Training, Beijing Sport University, Beijing, China; 2 Department of Sports Science and Clinical Biomechanics, SDU Sport and Health Sciences Cluster, University of Southern Denmark, Odense, Denmark; 3 China Institute of Sport Science, Beijing, China; 4 Department of Team Games, Wroclaw University of Health and Sport Sciences, Wrocław, Poland; 5 School of Sport Sciences, Faculty of Health Sciences, UiT, The Arctic University of Norway, Tromsø, Norway; 6 Danish Institute for Advanced Study (DIAS), University of Southern Denmark; 7 Sport and Health Sciences, College of Life and Environmental Sciences, University of Exeter, Exeter, United Kingdom

**Keywords:** Elite soccer, Internationaltournaments, Performance analysis, Goal scoring, Penalty shoot-out

## Abstract

Substitutions play a key role in modern football and can substantially affect the physical and overall performance of a team, and the recent substitution rule changes are worth investigating. This study explored the characteristics of substitutions, including different substitution rules, game results, sex, competition stages, tournaments and penalty shoot-outs success rates. We analysed data from a total of 3,738 substitutions from the last 10 years (2013–2023) of European Championships and World Cups, both men’s and women’s games. Non-parametric tests and chi-square tests were used for statistical analysis with the significance level set at p < 0.05. With the 5-substitution rule, 48% more substitutions occurred compared to the 3-substitution rule (4.26 ± 1.07 vs. 2.87 ± 0.43, p < 0.05) with a slight increase in the average substitution time (70.6 ± 14.3 vs. 69.2 ± 14.6 min, p < 0.05), and 10% more substitutions in the men’s game compared to the women’s game (p < 0.05). The timing of the first substitution was slightly different in the knock-out stage compared to group stage (59.8 ± 14.7 vs. 57.2 ± 13.3 min, p < 0.05), and the timing for the winning team and drawing team was later than for the losing team (p < 0.05). A total of 13.2% goals were scored by substitutes, with no significant difference between the 5-substitution rule (15.9%) vs the 3-substition rule (12.5%) (p > 0.05). Interestingly, substitute players had a lower success rate in penalty shoot-out compared to starters (61 vs. 74%, p < 0.05). Additionally, substitute player goal scorers entered the pitch later (p < 0.05) in male games compared to female games and in knock-out stage games compared to group games. This study highlights the importance of substitution rules and timing in modern elite football matches. The timing of the first substitution, introduction of substitutes in knock-out stages, and a lower success rate of substitute players in penalty shoot-outs are crucial factors to consider. Coaches can use this information to make strategic substitution decisions to improve team performance.

## INTRODUCTION

One of the most prevalent tactical adjustments made by football coaches during matches is substitutions which can be used for formation changes, rotation, or changes in style of play to achieve the coach’s intentions [1–3]. Additionally, starting players who are in poor condition but tactically important can be replaced by substitutes to maintain the physical performance of the team [4]. Therefore, the quality of the substitutes is often considered as a sign of the strength of a team [5, 6]. In 2020, Fédération Internationale de Football Association (FIFA) adopted the 5-substitution rule to ensure the fitness and health status of elite football players during and after the COVID-19 pandemic, and this rule has since been continued, possibly impacting the timing and number of substitutions in international men’s and women’s football. In light of this rule change and the increase in intensity of elite football matches [7, 8], Nassis and colleagues predict that substitutes will become even more important in football in the future and that practitioners will need to consider this more [9, 10]. In this situation, it is essential to study the characteristics of substitutions in modern football.

Using time-motion analysis, Bradley et al. [11] and Carling et al. [12] found that substitutes ran more at high intensity, both in terms of accumulated high-speed running as well as high-intensity running in peak intensity periods. Specifically in terms of high-speed running in 5-min periods, Hill et al. [13] found that the most intense 5 minutes of high-intensity running was when substitutes came on and Marcin et al. [14] found that the difference in sprinting distance between starters and substitutes was greatest at the very end of the game between 90–95 minutes. In addition, the running performance of substitutes is also affected by their position and stage of the season [15]. Despite the fact that the substitutes come on to the pitch with a greater running intensity, their overall game load is considerably lower than that of the starters due to the lower playing time [16, 17]. More recently, studies have shown that teams under the new substitution rule have an overall increase in running performance while the team load and RPE decrease [18–21], which is especially important during congested fixtures [22], indicating the importance of using multiple substitutions. In fact, the data from the 2022 World Cup shows that there was no reduction in high-intensity running by teams in the second half [23], in contrast to the results from previous World Cups [24].

When examining the tactical characteristics of substitutions, studies from La Liga [25] and Premier League [11] found that substitutions occurred mostly in the second half and regarding positional differences, most often for midfielders. Additionally, results from the aforementioned studies show that substitution time in La Liga is earlier than in the Premier League, and that the defensive players are replaced later in the game than the attacking players [25]. Specifically, the substitute player had an average playing time of 23–25 minutes in a French Ligue 1 teams [12], which means substitutions on average occur around 70 min, slightly earlier than in in the Premier League [11]. In addition, researchers found that losing teams used substitutions earlier in the game due to the need for tactical changes [25, 26], and had a better chance of changing the scoreline by using three substitutions before the 58^th^, 73^rd^ and 79^th^ minute, respectively [26]. Furthermore, late substitutions are sometimes used to make players available for penalty shoot-outs, and one study gave advice on prioritising substitute players for penalty shoot-outs [27].

Male and female football players have different physical capacity and physical match performance [28, 29]. With a lower physical capacity, especially anaerobic capacity, female athletes may experience fatigue earlier in the match compared to male players [30, 31]. Thus, coaches may adopt a different substitution strategy for female than male players. To the best of our knowledge, it has not yet been studied whether there is a difference between timing, number, or importance of substitutions in men’s and women’s elite football, and overall, the information about the impact of the 5-substitution rule is scarce. Nonetheless, recent data from domestic league football suggest men’s football has more high-intensity running under the 5-sub rule [19, 32], whereas this was not the case in a study on women’s football under the 5-sub rule [20]. However, compared to league matches, international tournament matches are more physically demanding for players due to the congested schedule [33]. In this case, a reasonable squad rotation and substitution is important for the overall performance of the team, especially for the most exploited players [34]. Therefore, a separate study of substitutions during tournaments is warranted.

Among the many football tournaments, the FIFA World Cup and the UEFA European Championship are the two highest-level tournaments. Thus, the aims of this study were to examine the substitution and goal scoring characteristics of the World Cups and Euros for male and female elite football players over the last 10 years in relation to different rules, gender, stages, tournaments, ordinary playing time and penalty shoot-outs.

## MATERIALS AND METHODS

### Sample

We analysed a total of 549 matches and 3,738 substitutions in the Women’s World Cup in 2015, 2019, 2023, the World Cup in 2014, 2018 and 2022, the Women’s UEFA Euro in 2013, 2017 and 2022 as well as the UEFA Euro 2016 and 2020. The men’s and women’s 5-substitutition rule matches accounted for 39.1% and 37.3% of the total number of analysed matches, respectively. All match data were taken from the official websites of FIFA (www.fifa.com) and UEFA (www.uefa.com) and transferred to the Excel format. As all data are anonymous and available online and no participants needed for the study, no ethical documentation was required.

### Procedure

Substitute data for different match results (win, draw and lose), phases (group and knock-out stage), sex (men and women) and rules are counted separately and analysed for comparison. Average substitution time was determined as the total sum of substitution times for all substitutes divided by the number of substitutes, and the first substitution time is based on the time the first substitute athlete come onto the pitch. When comparing average substitution time and the average number of playing minutes for the substitute players, extra time data were excluded to avoid data confusion [35, 36]. However, substitution goals in extra time were included in the goal characteristic analysis and the result after ordinary playing time was used as the match result. Substitution goals were furthermore categorised as goals with or without an impact on the outcome of the match based on whether the result of the game changed after removing the substitute goal. With relation to matches with extra time, statistics are merely done in relation to the participation of substitutes in penalty shoot-outs.

### Statistical analysis

Data were processed using SPSS 26.0 (IBM, Chicago, USA) software. Normality was evaluated using Kolmogorov-Smirnov tests. Data are expressed as mean and standard deviation. Differences in time and numbers between different results, gender, phases, rules, match impact and tournaments were analysed using the Mann-Whitney U test and Kruskal–Wallis test with Dunn’s post hoc tests as data was not normally distributed. Chi-square tests were used to analysis differences in the ratios of goals with regards to different substitutes and starters as well as men and women. The significance level was set at p < 0.05.

## RESULTS

Table 1 presents the analysis of variance for mean substitution time, first substitution time and number of substitutions. The results show that 48% more substitutions are made with the 5 vs. 3-substitution rule (4.3 ± 1.1 vs. 2.9 ± 0.4, z = -22.228, p < 0.05) and that the average substitution appeared 1.4 min later with the 5 vs. 3-substitution rule (70.6± 14.3 vs. 69.2 ± 14.6 min, z = -3.067, p < 0.05). No significant difference was found between gender, stages and tournaments (z = -1.116 to -1.534, p > 0.05). There was no significant difference in the time of first substitution by gender, rules and tournaments (z = -0.339 to -1.414, p > 0.05). However, a difference in the time of first substitution by stage was found (57.2 ± 13.3 vs. 59.8 ± 14.7 min, z = -2.564, p < 0.05), which shows a later time of first substitution in knock out stage. In addition, there are more substitution in men’s football (3.5 ± 0.7 vs. 3.2 ± 0.5, z = -4.953, p < 0.05) compared to women and the 3-substitution rule, but there is no difference between different stages and tournaments in terms of the number of substitutions (z = -0.027 to -1.571, p > 0.05).

**TABLE 1:** Comparison of substitution time and number of substitutions between different gender, rules and stages.

		Mean substitution time (min)	Z	p	First substitution (min)	Z	p	No. of substitutes (n)	Z	p
Gender	Men	70.0 ± 14.3	-1.116	0.264	58.0 ± 13.3	-0.761	0.447	3.54 ± 0.98	-4.953	0.000*

	Women	69.5 ± 14.6			57.8 ± 14.2			3.24 ± 1.00		

Rules	3-Substitution	69.2 ± 14.6	-3.067	0.002*	58.0 ± 13.9	-0.339	0.735	2.87 ± 0.43	-22.228	0.000*

	5-Substitution	70.6 ± 14.3			57.8 ± 13.5			4.26 ± 1.07		

Stages	Group	69.7 ± 14.4	-1.461	0.144	57.2 ± 13.3	-2.564	0.010*	3.39 ± 0.94	-0.027	0.978

	Knock out	70.3 ± 14.5			59.8 ± 14.7			3.44 ± 1.16		

Tournaments	World Cup	69.5 ± 14.6	-1.534	0.125	57.6 ± 13.8	-1.434	0.152	3.36 ± 0.95	-1.571	0.116

	Euro	70.4 ± 14.1			58.5 ± 13.7			3.49 ± 1.10		

* denotes significant difference between groups

**FIG. 1:**
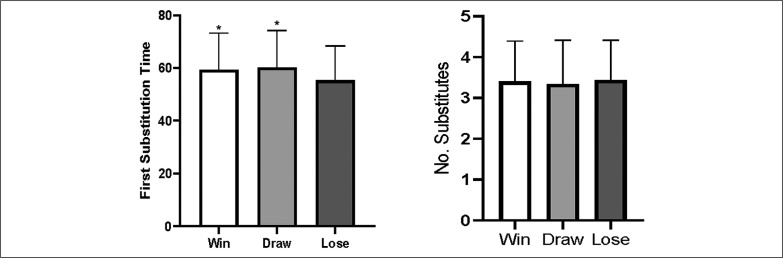
Substitutions characteristics in relation to the match outcome.

With regard to match outcome, there was a significant difference in the time of first substitution (k = 33.773, p < 0.05, figure 1), with winning and draw teams made their first substitution later compared to losing teams (59.3 ± 13.9, 60.2 ± 14.1 vs. 55.3 ± 13.1 min, p < 0.05). For the number of substitutes, no difference between match outcomes were observed (k = 1.083, p > 0.05).

**FIG. 2:**
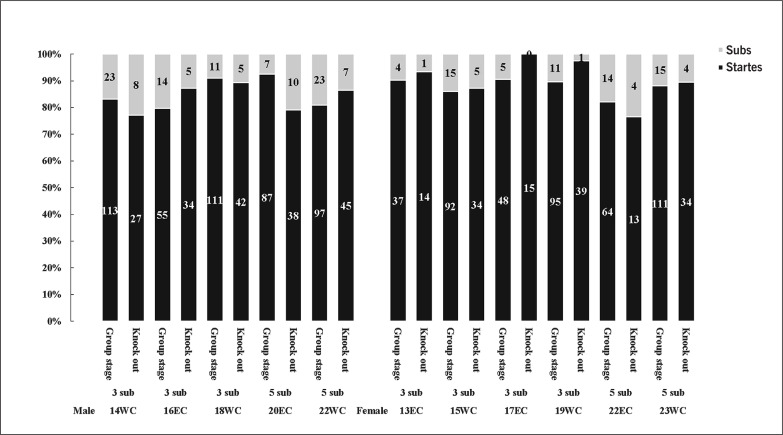
Goal distribution between starters and substitute players during the 2013-2023 World Cups and European Championships in men’s and women’s football.

Figure 2 shows the general goal distribution during the last 10 years’ WC and EC. The percentage of goals scored by substitutes in the last 10 years of the EC and World Cup is 13.4%, with no significant differences found in the percentage of substitute goals scored by male and female footballers (14.8 vs. 11.7%, χ^2^ = 3.021, p > 0.05), 5 and 3-substitute rules (14.7 vs. 12.5%, χ^2^ = 1.388, p > 0.05), different stages (13.5 vs. 13.0%, χ^2^ = 0.055, p > 0.05) and tournaments (13.2 vs. 13.6%, χ^2^ = 0.049, p > 0.05, table 2). Interestingly, the success rate of substitute players in penalty shoot-outs was observed to be significantly lower compared to the starters (61.4 vs. 73.9%, χ^2^ = 5.019, p < 0.05).

**TABLE 2:** Goal characteristics comparison of starters and substitutes

		Substitutes	Starters	χ^2^	P
Gender
	Men	113	649	3.021	0.082

	Women	79	596		

Rules
	3-substitution	108	756	1.388	0.239

	5-substitution	84	489		

Stages
	Group	142	910	0.055	0.814

	Knock out	50	334		

Tournaments
	World Cup	128	840	0.049	0.825

	Euro	64	405		

Penalty shoot-outs
	Success	70	130	5.019	0.025*

	Failed	44	46		

Data presented as number of goals (n),

* denotes significant difference between groups

The chi-square analysis shows no significant differences (χ^2^ = 0.519–3.468, p > 0.05) between male and female athletes in terms of goal contributions by substitutes in different stage, rules and match impact. However, the p value and percentages indicate a trend towards more substitutes’ goals for men’s football during knock-out stage (31.0 vs. 19.0%), with a trend towards a larger impact on results (54.0 vs. 41.3%, Table 3) compared to women’s football (p < 0.10).

**TABLE 3:** Comparison of Substitutes Goal Characteristics for men’s and women’s football tournaments.

		Men	Women	χ^2^	P
Rules	3-substitution	66	42	0.519	0.471

	5-substitution	47	37		

Stages	Group	78	64	3.468	0.063

	Knock out	35	15		

Impact on match results	Yes	61	33	3.039	0.081

	No	52	47		

Data presented as number of goals (n).

**FIG. 3:**
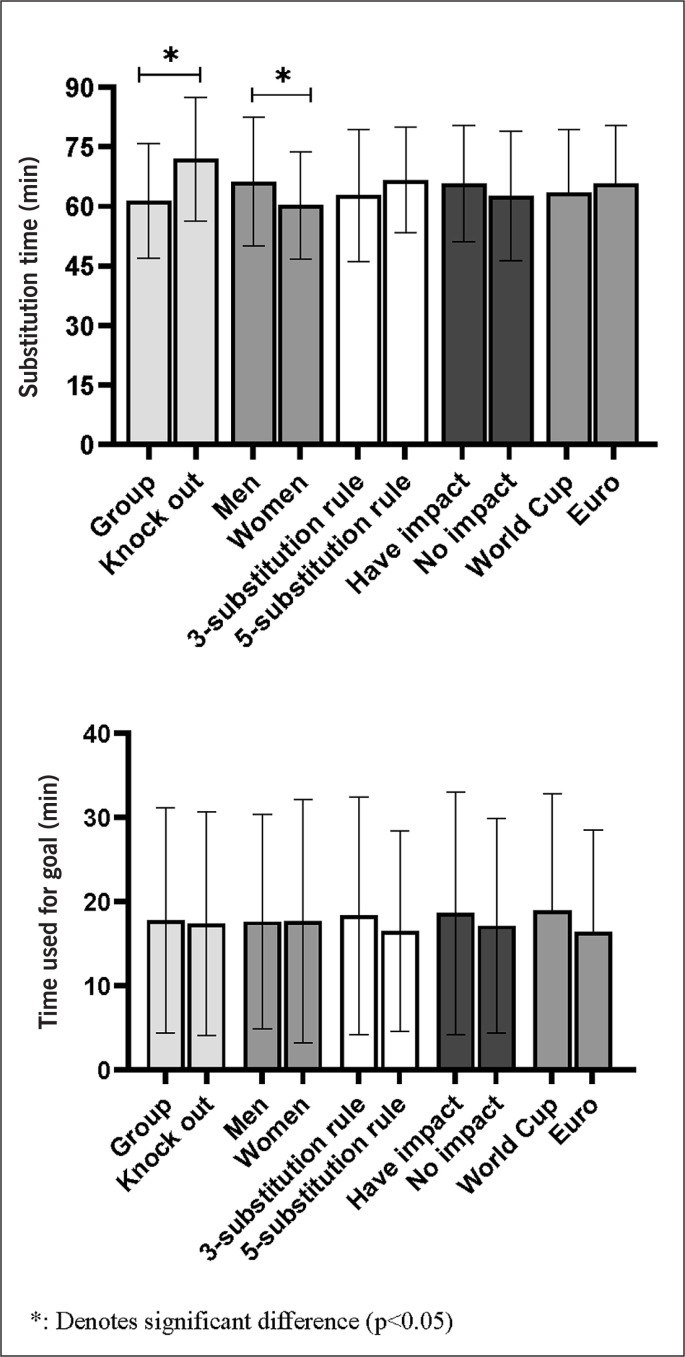
Goal scoring characteristics for substitute players in relation to substitution time and time on the pitch during the 2013-2023 World Cups and European Championships in men’s and women’s football.

Substitutes who scored a goal came on the pitch later in the men’s games than the women’s games (66.3 ± 16.1 vs. 60.3 ± 13.5 min, z = -2.404, p < 0.05, figure 3), and during knockout stage compared to group stage (61.4 ± 16.1 vs. 71.9 ± 13.5 min, z = -3.695, p < 0.05), while there was no difference across rules, match results impact and tournament (z = -0.780 to -1.401, p > 0.05). The goalscoring substitute players were substituted 18.1 ± 13.4 minutes prior to the goal scoring situation, with no differences between rules, gender, stages, result impacts and tournaments (z = -0.017 to -1.108, p > 0.05).

## DISCUSSION

The present study analysed the characteristics of substitutions in top-level tournaments for male and female players under different substitution rules, and revealed a marked increase in the number of substitutions and substitution playing minutes with the introduction of the 5-substitution rule. Moreover, it was shown that more substitutions are performed in the men’s compared to the women’s game, that the fraction of goals scored by substitute players has reached ~15% and that substitute players scoring goals came on later in the knockout games and in the men’s game. Interestingly, the scoring rate in penalty shoot-outs was observed to be much lower for substitute players compared to starters.

The average time of substitutions in our study is around 70 min, which is in line with previous studies of French Ligue 1 [12] and Premier League [11], but later than in La Liga [25], with 10% more substitutions in the men’s game compared to the women’s game. This is somewhat surprising as fatigue is just as predominant in women’s elite football as in men’s football [31], and some studies indicate that fatigue occurs earlier in the women’s game [37]. On the other hand, it may be speculated that the female substitutes are less capable than starters, especially in lower ranked teams. This is supported by tendencies towards a higher percentage of goals scored that affected the outcome of the game by men’s substitutes than women’s substitutes (54% vs 41%, NS, p = 0.081) as well as goals scored in the knock-out stage compared to the group stage (31 vs 19%, NS, p = 0.063). One clear exception from this finding was seen in the Women’s Euro 2021 where the England team performed early substitutions in all games and scored 8 substitute goals in the tournament, including both goals in the final against Germany. Future studies are required to investigate the gender differences in greater depth.

No difference was observed between the 5 and 3-substitution rules in terms of time for the first substitution. However, the average substitution time was significantly later with the 5-substitution rule, as coaches used a larger percentage of the 5 substitutes in the final stages of the game. The first substitution may be more influenced by the team’s performance, and a very early substitution may be seen as a substitution for a player in poor physical condition or as a tactical mistake by the coaching staff. However, the “cost” of a substitution is reduced with five substitutions making it easier for the coaching to consider early substitutions. In present study, it was shown that the first substitution was conducted later in the knock-out stage compared to the group stage. Previous studies from from World Cups have indicated that the group stage has different tactics compared to the knockout stage [36, 38]. Considering that many knock-out matches continue to the extra time and penalty shoot-out, the coaches would thus be more cautious using substitutions in ordinary playing time. Another finding in this study regarding the timing of the first substitution was that the losing team substituted earlier than the draw and winning team, which is consistent with previous studies [25, 26]. The score line has been found to be one of the most critical factors influencing substitutions [5, 39]. With the score trailing, the coaches were more likely to adjust their tactics in order to break the passive situation on the field and try to tie the score [39].

When examining the goals scored by substitutes, we found that substitutes scored 14% of all goals, of which around 50% directly influenced the outcome of the game. The substitute players accounted for around 9–10% of the playing time, meaning that substitute players had a higher goal per playing time score than the starters. Considering that substitutes can also impact the game in other ways such as through challenges, assists, defending and improved running performance [21], it is important for coaches to arrange substitutions thoughtfully. Contrary to our hypothesis, no significant differences were found in the percentage of goals scored by substitutes during matches with different substitution rules, albeit we observed some statistical tendencies. The reasons for this may be that only 36% of the tournaments in this study applied the 5-substitution rule; goals were affected in a number of ways and averaging less than 3 goals a game. Another more important reason is that teams have a limited number of strikers and coaches cannot have all their substitutions in the offensive line at the expense of the defence. In fact, most of the starting players were replaced by players from the same playing position [4, 39]. Furthermore, the aforementioned data suggests that the average substitution happens later in the matches with the 5-substitution rule, and the later the substitution, the more likely it is to happen for a defensive players [25], which could mean that defensive substitutes account for a higher fraction of the substitutions under the 5-substitutition rule. This means that the 5-subsitution rule may lead to a higher overall running performance [19, 32], but this change is related to the number as well as the type of substitutions being made [20]. In addition, coaches may use substitutions to reduce the load of team [18, 21], which is extremely important to maintain the physical condition of the whole team in the World Cup with a congested schedule.

Interestingly, we found that substitutes score less often than the starting players in penalty shoot-outs (61.4 vs. 73.9%), with an almost identical difference for male players (61.4 vs. 73.8%) and female players (61.3 vs. 73.9%). We also observed more penalties taken by substitute players in the penalty shoot-outs in the men’s tournaments compared to the women’s tournaments (43.7 vs 31.0%), leading to an overall scoring percentage of 68.4% in the men’s tournaments and 70.0% in the women’s tournaments. Considering that the number of substitute players taking penalties have more than doubled over the last two decades coming from 2 to 3 to 3+1 to 5+1 substitutions in the international tournaments, this may have a decisive role in the distribution of medals, as seen in the Euro 2022 men’s final, where England’s two last-minute substitute players both missed their penalties. Penalty shoot-outs are extremely mentally demanding for players [40, 41], and without good mental preparation, it appear to be difficult to achieve success in penalty shoot-outs [41]. In fact, substitutes are usually under more pressure and anxiety than the starting athletes [42, 43]. If a player is substituted into the game, the fear of failure may be even more pronounced [42], although it may be considered an advantage that the substitute players are less fatigued. Another reason may also be that, on average, substitutes may be less experienced than starters [16, 44]. Furthermore, there is a greater psychological burden on players who are substituted exclusively for taking part in the penalty shoot-outs. More research is required to elucidate possible explanations for the lower penalty success rate of substitute players. Nonetheless, coaches need to be careful in their selection of players for the penalty shoot-out and be aware that the substitute players appear to have a higher miss rate in penalty shoot-outs in international tournaments.

Finally, we found that substitute goal scorers came on later in the knockout stage than in the group stage and among male compared to female players. The main reason why substitute goal scorers come on later in the knockout stages may be that coaches want more fresh players in the extra time to improve the chances of success in the decisive part of a the long and fatiguing games, which may also explain the statistical tendencies of the higher level of substitute goals being scored in the knockout phase. Aside from this, no difference was found between different gender, rules, stages and impact on matches in terms of time taken to score. However, we found that the highest frequency of substitute goals was 2 minutes after they come onto the pitch. More goals scored during this period may be because the defenders had not yet clarified their defensive responsibilities in relation to the new players coming in, and that the substitute players have their peak 5-min high-intensity running period in the first five minutes after substitution [13].

One limitation of this study is that the 5-substition rule is new and that relatively limited data are available under the new substitution rule, and that coaches have adapted gradually to the new substitution possibilities. In the future, more match data under the 5-substitution rule will be available, which will enable us to explore the full impact of the 5-substitution rule on the impact on the modern elite football game and find the interaction effect of multiple two variables in relation to the substitution characteristics. Moreover, it should be emphasized that this study solely investigated international tournaments and that specific research on the role of substitute players in the domestic leagues should be studied separately.

## CONCLUSIONS

Taken together, we observed a huge increase in the number of substitutions and substitution playing minutes with the introduction of the 5-substitution rule, and that that the fraction of goals scored by substitute players have reached ~15%. It was also seen that substitute players scoring goals came on later in the knockout games compared to the group stage, and that more substitutions are currently performed in the men’s compared to the women’s game. Another interesting new finding of the present study was that the scoring rate in penalty shoot-outs was much lower for substitute players compared to starters, both in international men’s and women’s tournaments. Thus, the present study provides coaches with valuable information on the characteristics of substitutions in modern international men’s and women’s elite football tournaments, which may enable them to make better decisions about when to make substitutions and on selecting the right players to use in penalty shoot-outs, potentially leading to better game outcomes.
